# Golexanolone, a GABA_A_ receptor modulating steroid antagonist, restores motor coordination and cognitive function in hyperammonemic rats by dual effects on peripheral inflammation and neuroinflammation

**DOI:** 10.1111/cns.13926

**Published:** 2022-07-26

**Authors:** Gergana Mincheva, Carla Gimenez‐Garzo, Paula Izquierdo‐Altarejos, Mar Martinez‐Garcia, Magnus Doverskog, Thomas P. Blackburn, Anneli Hällgren, Torbjörn Bäckström, Marta Llansola, Vicente Felipo

**Affiliations:** ^1^ Laboratory of Neurobiology Centro de Investigación Príncipe Felipe Valencia Spain; ^2^ Umecrine Cognition AB Solna Sweden; ^3^ Umeå Neurosteroid Research Center Clinical Sciences at Umeå University Umeå Sweden

**Keywords:** GR3027, inflammation, minimal hepatic encephalopathy, motor incoordination, spatial memory

## Abstract

**Aims:**

Hyperammonemic rats show peripheral inflammation, increased GABAergic neurotransmission and neuroinflammation in cerebellum and hippocampus which induce motor incoordination and cognitive impairment. Neuroinflammation enhances GABAergic neurotransmission in cerebellum by enhancing the TNFR1‐glutaminase‐GAT3 and TNFR1‐CCL2‐TrkB‐KCC2 pathways. Golexanolone reduces GABA_A_ receptors potentiation by allopregnanolone. This work aimed to assess if treatment of hyperammonemic rats with golexanolone reduces peripheral inflammation and neuroinflammation and restores cognitive and motor function and to analyze underlying mechanisms.

**Methods:**

Rats were treated with golexanolone and effects on peripheral inflammation, neuroinflammation, TNFR1‐glutaminase‐GAT3 and TNFR1‐CCL2‐TrkB‐KCC2 pathways, and cognitive and motor function were analyzed.

**Results:**

Hyperammonemic rats show increased TNFα and reduced IL‐10 in plasma, microglia and astrocytes activation in cerebellum and hippocampus, and impaired motor coordination and spatial and short‐term memories. Treating hyperammonemic rats with golexanolone reversed changes in peripheral inflammation, microglia and astrocytes activation and restored motor coordination and spatial and short‐term memory. This was associated with reversal of the hyperammonemia‐enhanced activation in cerebellum of the TNFR1‐glutaminase‐GAT3 and TNFR1‐CCL2‐TrkB‐KCC2 pathways.

**Conclusion:**

Reducing GABA_A_ receptors activation with golexanolone reduces peripheral inflammation and neuroinflammation and improves cognitive and motor function in hyperammonemic rats. The effects identified would also occur in patients with hepatic encephalopathy and, likely, in other pathologies associated with neuroinflammation.

## Graphical abstract

Immunotherapy has emerged as a promising treatment for GBM patients. The impact of several molecular and clinical variables on survival outcomes of GBM patients treated with immunotherapy has been comprehensively assessed in this systematic review and meta‐analysis. Our findings might help to optimize the immunotherapy efficacy for GBM patients and give insights into grouping patients in clinical trials of immunotherapy for GBM patients.

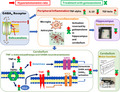

## 1 INTRODUCTION

Several million patients with liver cirrhosis suffer minimal hepatic encephalopathy (MHE), with mild cognitive impairment and motor incoordination, which reduces the quality of life and increases the risk of accidents and hospitalizations, thus imposing heavy costs on health systems.^1^, ^2^, ^3^, ^4^, ^5^, ^6^, ^7^

Treatment of MHE may improve cognitive and motor function in MHE patients. Rifaximin is a non‐permeable antibiotic approved for preventing hepatic encephalopathy (HE) appearance in cirrhotic patients.^8^ Rifaximin treatment improves neurological function in 60% of MHE patients, but not in the remaining 40%.^9^ There are no specific treatments for the neurological alterations in MHE and clinical HE and new, more effective treatments, acting on the mechanisms that induce cognitive and motor impairments are needed.

Hyperammonemia and peripheral inflammation induce neurological impairment in patients and animal models.^10^, ^11^, ^12^, ^13^, ^14^, ^15^ Hyperammonemic rats reproduce the cognitive and motor alterations of MHE patients. Hyperammonemia induces peripheral inflammation, which induces neuroinflammation. Cognitive impairment is mainly due to neuroinflammation‐induced alterations in glutamatergic neurotransmission in hippocampus.^14^, ^15^, ^16^, ^17^, ^18^ Motor incoordination is mainly due to neuroinflammation‐induced increase in GABAergic neurotransmission in the cerebellum. Hyperammonemia induces microglia and astrocytes activation and increases TNFα and membrane expression of the TNFα receptor TNFR1 in the cerebellum. Increased activation of TNFR1 enhances GABAergic neurotransmission by activating the TNFR1‐glutaminase‐GAT3 pathway and the TNFR1‐CCL2‐TrkB‐KCC2 pathway.^14^, ^19^, ^20^

Enhanced GABAergic neurotransmission induces motor incoordination in hyperammonemia and MHE. A plausible therapeutic approach to improve cognitive and motor function would be treatments aiming at reducing GABAergic neurotransmission. Reducing GABAergic neurotransmission using the GABA_A_ receptor antagonist bicuculline or with the neurosteroid pregnenolone sulfate improves cognitive and motor impairments in hyperammonemic rats.^21^, ^22^, ^23^, ^24^, ^25^ However, the use of antagonists of GABA_A_ receptors would not be useful in clinical practice. A more useful approach would be to reduce GABAergic neurotransmission by using compounds that modulate indirectly GABA_A_ receptors. Allopregnanolone (3a‐hydroxy‐5a‐pregnane‐20‐one) is a positive allosteric modulator of GABA_A_ receptors which is increased in the brain of hyperammonemic rats^25^ and of cirrhotic patients who died in hepatic coma,^26^ and may enhance GABAergic neurotransmission. Reducing the potentiation of GABA_A_ receptors activation by allopregnanolone could be a safe way to reduce GABAergic neurotransmission. Golexanolone (GR3027), a novel investigational drug in clinical development, is a GABA_A_ receptor modulating steroid antagonist which reduces the potentiation of GABA_A_ receptors by allopregnanolone in animal models and in humans and is a promising therapeutic tool to improve cognitive and motor function in hyperammonemia and HE.

Repeated subcutaneous injections of golexanolone, at doses generating plasma levels safe in animal toxicology studies, restores motor coordination and cognitive function in hyperammonemic rats, a model of MHE,^27^ and improves cognitive performance in a pilot phase 2a study in patients with MHE.^28^ However, the mechanisms involved have not been fully elucidated.^27^, ^28^

There is an interplay between GABAergic neurotransmission and neuroinflammation, which modulate each other, and contribute to induction of cognitive and motor impairment.^14^, ^29^ It would be therefore possible to reduce neuroinflammation by reducing GABAergic neurotransmission. We hypothesized that the beneficial effects of golexanolone on cognitive and motor function in hyperammonemic rats are not only due to its direct action on GABAergic neurotransmission, but golexanolone would also reduce neuroinflammation, which would contribute to reducing GABAergic neurotransmission by reducing the activation of the TNFR1‐glutaminase‐GAT3 pathway and the TNFR1‐CCL2‐TrkB‐KCC2 pathway.

Treatment of hyperammonemic rats with bicuculline reduces peripheral inflammation.^21^ Golexanolone could also reduce peripheral inflammation, which may contribute to reducing neuroinflammation and improving cognitive and motor function.

The aims of this study were to: (1) assess if golexanolone reduces neuroinflammation in cerebellum and hippocampus of hyperammonemic rats, and if it reduces microglia and/or astrocytes activation; (2) identify mechanisms by which golexanolone may reduce GABAergic neurotransmission in cerebellum: analyze the effects on the TNFR1‐glutaminase‐GAT3 pathway and the TNFR1‐CCL2‐TrkB‐KCC2 pathway; (3) assess if golexanolone improves peripheral inflammation by analyzing TNFα, IL‐10 and TGFβ. In our previous study with golexanolone, the compound was administered by subcutaneous injection.^27^ As this is not a convenient administration route in clinical practice, we have developed a formulation which may be administered orally. We also aimed to assess if intragastric administration of golexanolone improves cognitive and motor function in hyperammonemic rats.

## 2 METHODS

### 2.1 Animal model and treatment

**FIGURE 1:**
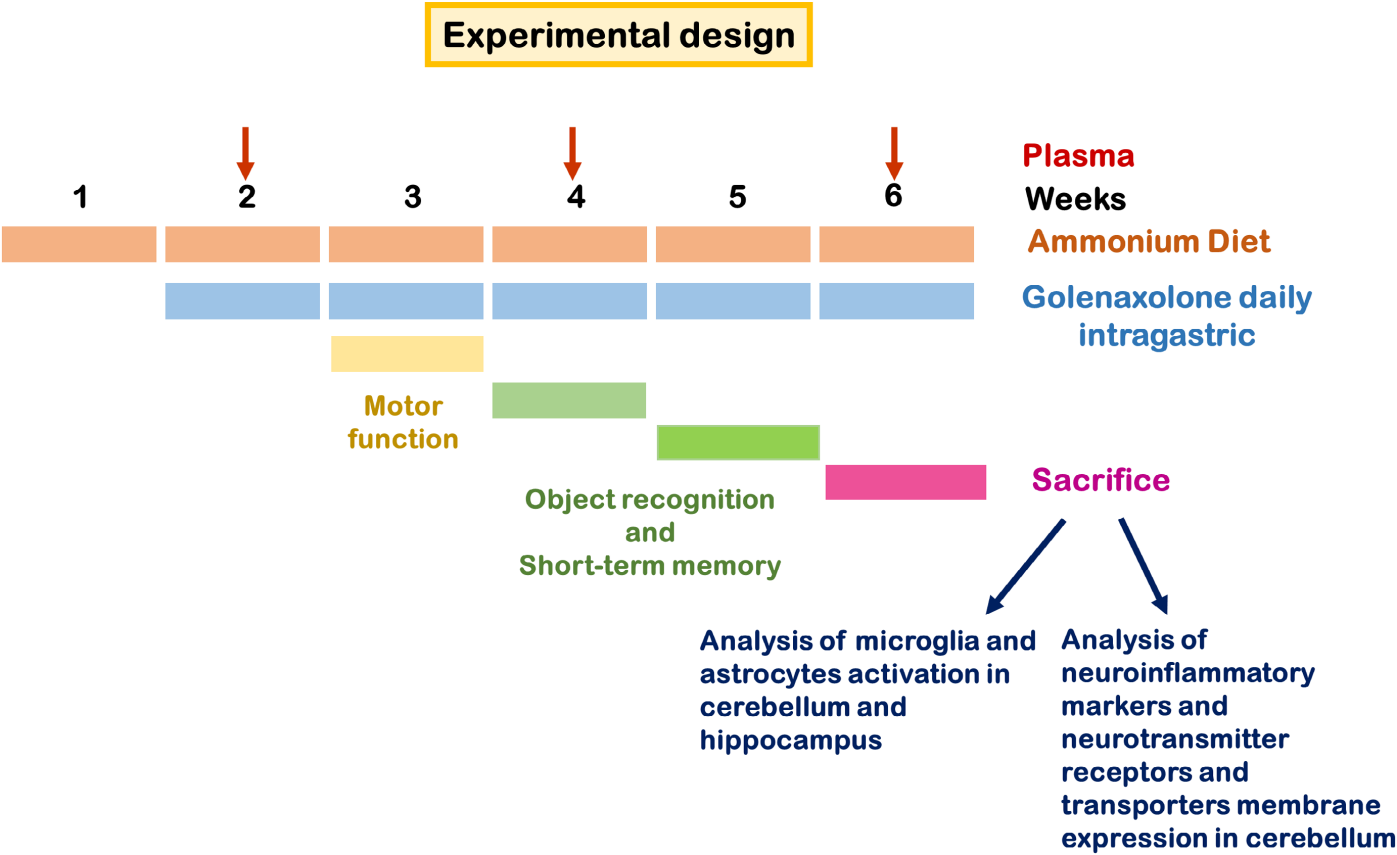
Scheme showing the experimental design

Forty‐eight male Wistar rats (220–250 g) were used. Rats were made hyperammonemic by feeding an ammonia‐containing diet as made by Felipo et al.^30^ The experimental design is shown in Figure 1. After 1 week of ammonia‐containing diet, rats were divided in four groups of twelve rats: controls treated with vehicle (CV), controls treated with golexanolone (CGR); hyperammonemic‐vehicle (HV) and hyperammonemic rats treated with golexanolone (HGR). Golexanolone (40 mg/ml) in CAPMUL (Capmul MCM, EP, ABITEC, USA) was administered daily through an intragastric plastic tube (Instech, USA) at 50 mg/kg (1.25 ml/kg) during five weeks. An equivalent volume of CAPMUL was administrated to controls. The experiments were approved by Comité Ético de Experimentación Animal (CEEA) of our center and by Conselleria de Agricultura, Generalitat Valenciana, and performed according to the Directive of the European Commission (2010/63/EU) for the care and management of experimental animals and complied with the ARRIVE guidelines for animal research.

### 2.2 Safety and systemic exposure

A 6‐month GLP‐compliant toxicity study was performed as part of the documentation for clinical trials. Twelve male and 12 female Wistar rats per dose group were dosed once daily with up to 100 mg/kg/day of golexanolone. In addition, six male and six female rats per dose group were dosed similarly to the main animals and sampled for assessment of systemic exposure to golexanolone. Plasma samples were analyzed using a LC–MS/MS method. The study was performed by Envigo CRS, Spain. The results from this study were used to determine the dose for the animal model described above.

### 2.3 Motor function

Motor function: motor coordination and gait parameters was assessed after two weeks of golexanolone administration.

#### 2.3.1 Footprint analysis of locomotor gait in the CatWalk™

This is a video‐based automated gait analysis system (Noldus, Wageningen, The Netherlands). Three trials were recorded each day during two days. Gait analysis values are the mean of six runs. Data were analyzed using the CatWalk analysis software (v 7.1).^31^

#### 2.3.2 Motorater

A kinematic analysis of motor coordination was conducted using MotoRater apparatus (TSE Systems, Germany) as in Ref. [32]. Each day three uninterrupted runs were recorded for each rat, over three days. The runs were analyzed by counting and classifying the steps as correct or wrong paw placements. The results are expressed as percentage of total steps and are the mean of nine runs.

### 2.4 Cognitive function assessment

#### 2.4.1 Novel object recognition (NOR) and novel object location (NOL) memory tests

Tests were performed in an open‐field arena with visuospatial cues on the walls as in Ref. [33]. The NOL test was performed on day six. The NOR test was performed on day seven. A discrimination ratio was calculated as the difference between the times spent exploring the object whose location had been changed (NOL) or the new object (NOR) with the unchanged object divided by total time exploring.

#### 2.4.2 Short‐term spatial recognition memory

Short‐term spatial recognition memory was analyzed using a Y‐maze. The rat was placed into one arm (start arm) and allowed to explore the maze with one arm closed, for 2 min (training trial) two times, with 1 min of inter‐trial interval. Then, the rat was allowed to explore all three arms for 2 min (test trial). The number of entries into and the time spent in each arm were registered and the discrimination ratio [(time spent in the novel arm–time spent in the familiar arm)/total time passed in the two arms] was calculated.

### 2.5 Immunohistochemistry

Rats (six per group) were anesthetized with sodium pentobarbital and transcardially perfused with 0.9% saline followed by 4% paraformaldehyde in 0.1 M phosphate buffer (pH 7.4). Brains were removed and post‐fixed in the same fixative solution for 24 h at 4°C. Five‐micrometer thick, paraffin‐embedded sections (5 μm) were cut and mounted on coated slide glass Primary antibodies used for the study were: anti IBA1 (Wako 019‐19741); 1:300 for 30 min, anti GFAP (SIGMA). Sections were counterstained with Mayer's hematoxylin (DAKO S3309) for 5 min.

### 2.6 Analysis of astrocytes and microglia activation

Sections were scanned with an Aperio Versa system (Leica Biosystems, Germany). Fields at 40× magnification were captured using the software ImageScope64; 8–10 images per rat were taken from three different sections of the hippocampus or the cerebellum. Microglial activation was analyzed by measuring the area of Iba1 stained cells with IpWin 32 software program and astrocytic activation by measuring the GFAP stained area with ImageJ software as in Ref. [34]

### 2.7 Analysis of protein content by Western blot

After five weeks of golexanolone treatment, cerebellum and hippocampus were dissected from six rats per group and homogenized in 50 mM TRIS–HCl pH 7.5, 50 mM NaCl, 10 mM EGTA, 5 mM EDTA and protease and phosphatase inhibitors. Thirty μg of protein was loaded in a 15% SDS gel and immunoblot was performed with antibodies against: GFAP (Sigma, 1:5000), IBA1 (Abcam, 1:500), CCL2 (Proteintech, 1:200), TNF‐a (R&D SYSTEMS, 1:500), Glutaminase (Novus, 1:1000), GAT‐3 (Abcam, 1:1000), TrkB (Abcam, 1:500), GAD67 (Abcam, 1:500) and GABA_A_ beta3 subunit (Abcam, 1:1000). β‐Actin (Abcam, 1:5000) or GAPDH (Millipore, 1:15,000) were used as a control for protein loading. For plasma proteins analyzed by Western blot we used Coomassie R‐350 staining on the membrane, after immunoblotting,^35^ as loading control. We selected a Coomassie‐stained band that was not saturated as representative of the whole protein lane. For quantification, intensity of plasma proteins was divided by intensity of complete protein lane, as is performed in Ref. [35].

### 2.8 Membrane expression of receptors and transporters

This was analyzed as in Ref. [36]. Transversal 400 μm thick cerebellar slices were added to tubes containing ice‐cold Krebs buffer with or without 2 mM BS3 (Pierce, Rockford, IL) and incubated for 30 min at 4°C. Cross‐linking was terminated by adding 100 mM glycine (10 min, 4°C). The slices were homogenized in lysis buffer (66 mM Tris–HCl pH 7.4. 1% SDS, 1 mM EGTA, 10% glycerol, 0.2 mg/ml leupeptin, 1 mM NaF, 1 mM Na‐orto‐vanadate) by sonication for 20 s. Samples treated with or without BS3 were analyzed by Western blot using antibodies against TNFR1 (Abcam, 1:1000), P2X4 (Invitrogen, 1:500), TrkB (Abcam, 1:500), KCC2 (Millipore, 1:1000) and GAT‐3 (Abcam, 1:1000). The surface expression of these proteins was calculated as the difference between the intensity of the bands without BS3 (total protein) and with BS3 (non‐membrane protein).^37^

### 2.9 Statistical analysis

Data are expressed as mean SEM. All statistical analyses were performed using GraphPad Prism software v. 9.0. Data were tested for normality (Kolmogorov–Smirnov or D'Agostino and Pearson test) and for homogeneity of variances. Statistical analysis was carried out using one‐way ANOVA and Tukey's multiple comparisons test or two‐way ANOVA with repeated measures when appropriate, and Bonferroni's multiple comparisons test. When data did not pass the normality test, the nonparametric Kruskal–Wallis test, with Dunn's test for multiple comparisons, was used. When standard deviations (SDs) were not equal, Welch's ANOVA was used.

## 3 RESULTS

Based on a chronic toxicity study in rats, a dose of 50 mg/kg/day was selected for the assessment of the effects of orally administered golexanolone. At this dosage, systemic exposure is expected to be in a range that is achievable, and has been tested in healthy volunteers and HE‐patients. Moreover, no toxicity was observed at 100 mg/kg/day, which was the highest dose tested.

**FIGURE 2:**
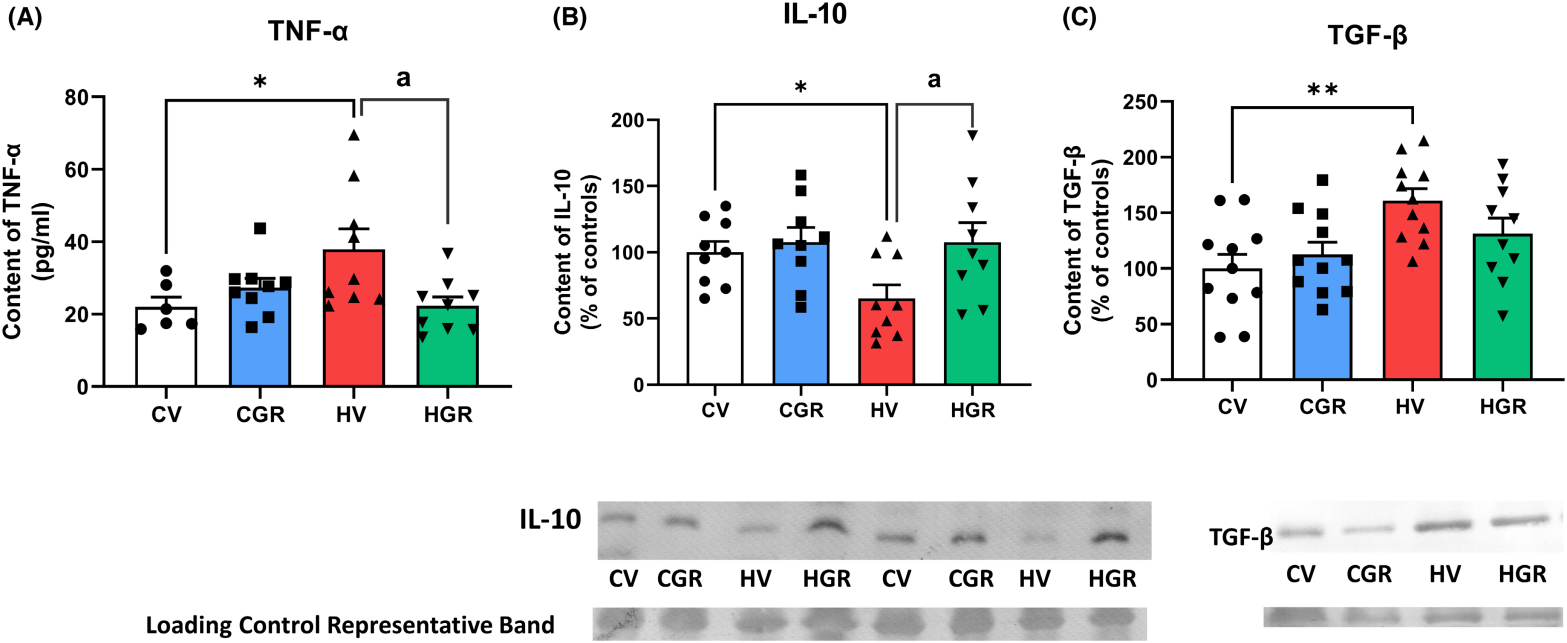
Golexanolone treatment reduces peripheral inflammation in hyperammonemic rats. TNF‐a in plasma was analyzed by ELISA (A). IL‐10 (B) and TGFb (C) in plasma were analyzed by Western blot. Values are the mean ± SEM of 9 rats per group in (A and B) and of 11 rats per group in C. Values significantly different from control rats are indicated by asterisk and from hyperammonemic rats by “a”. *p < 0.05; **p < 0.01; ^a^ *p* < 0.05. CGR, control group with golexanolone treatment; CV, control rats with treatment vehicle (CAPMUL); HGR, hyperammonemic rats with golexanolone treatment; HV, hyperammonemic rats with vehicle

We assessed if golexanolone reduces peripheral inflammation in hyperammonemic rats. Golexanolone reversed (22 ± 2 pg/ml) the increase of TNFα in plasma (38 ± 6 pg/ml in hyperammonemic rats and 22 ± 3 pg/ml in controls) from hyperammonemic rats (Figure 2A). The antiinflammatory interleukin IL‐10 was reduced in hyperammonemic rats (69 ± 13%) and was normalized by golexanolone (112 ± 11%) (Figure 2B). Hyperammonemic rats also show increased TGFβ (157 ± 9%) which was partially reduced by golexanolone (133 ± 12%) (Figure 2C).

**FIGURE 3:**
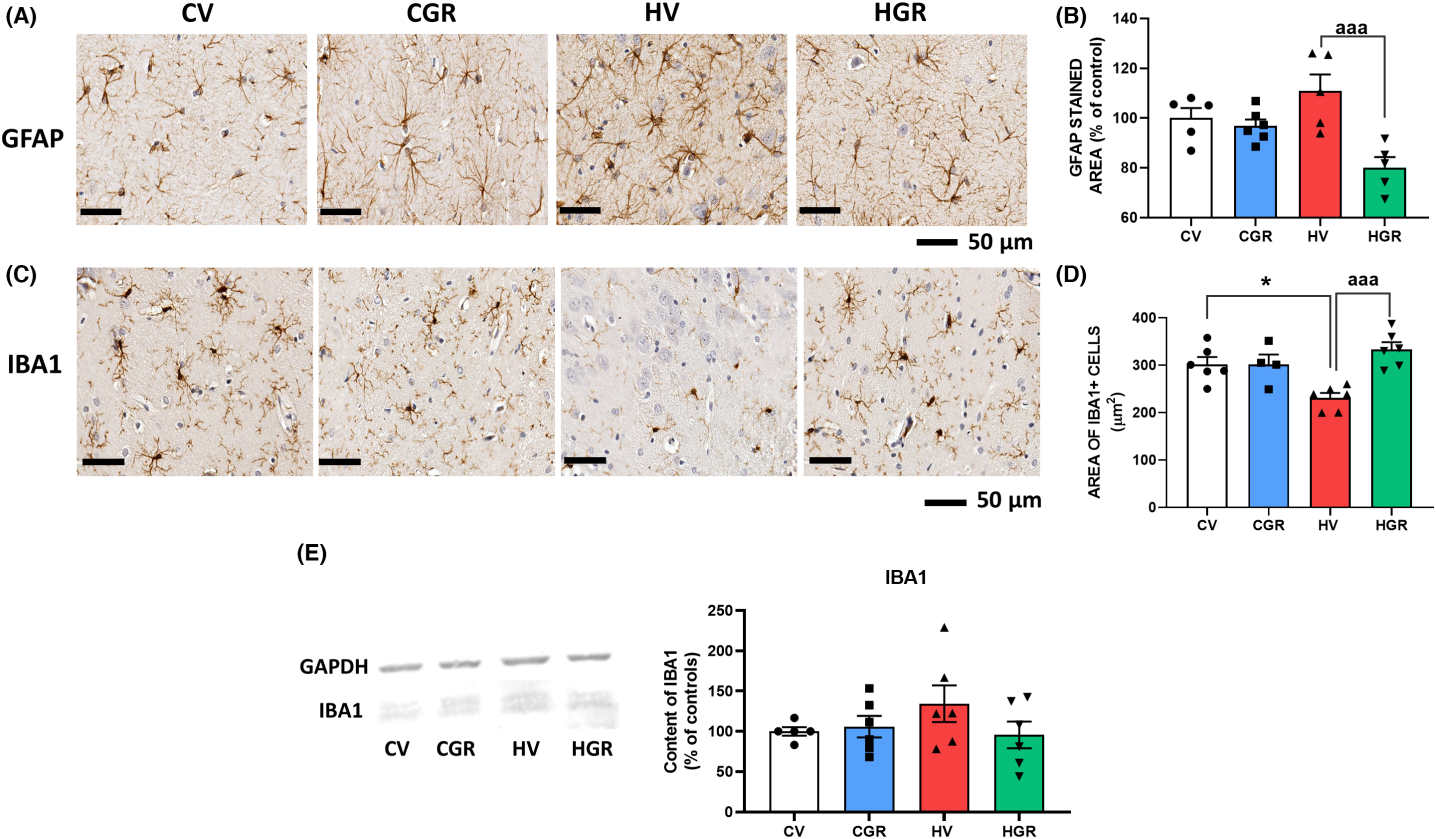
Golexanolone treatment reduces activation of astrocytes and microglia in hippocampus of hyperammonemic rats. Representative immunohistochemistry images of GFAP staining are shown in (A). The area stained by anti‐GFAP was quantified in whole hippocampus in two slides/rat from 6 rats per group. Values are expressed as percentage of stained area in control rats (B). Hippocampal microglia was stained with anti‐Iba1. Representative images are shown in (C). Microglia activation was quantified by measuring the area of Iba1^+^ cells (D). Content of Iba1 was analyzed by western blot in whole hippocampus homogenates. Representative bands and quantification is shown in E. Values are the mean ± SEM of 6 rats per group. Values significantly different from control rats are indicated by asterisk and from hyperammonemic rats by “a”. *p < 0.05; ^aaa^ *p* < 0.001. CGR, control group with golexanolone treatment; CV, control rats with treatment vehicle (CAPMUL); HGR, hyperammonemic rats with golexanolone treatment; HV, hyperammonemic rats with vehicle

Hyperammonemic rats show a mild increase in GFAP staining indicating mild astrocytes activation in hippocampus. Treatment with golexanolone reduced the GFAP staining in hyperammonemic rats (Figures 3A,B and S1) (111 ± 7% in hyperammonemic rats vs. 81 ± 4% in hyperammonemic rats treated with golexanolone) indicating that golexanolone reduces astrocytes activation.

Microglia was activated in hippocampus of hyperammonemic rats as indicated by a reduced area of Iba1 marked cells. Golexanolone reversed this reduction (231 ± 10 μm^2^ in hyperammonemic rats vs. 333 ± 15 μm^2^ in hyperammonemic rats treated with golexanolone, and 290 ± 13 μm^2^ in controls) indicating reversal of microglia activation (Figures 3C,D and S2). Western blot analysis of Iba1 content show that total content of Iba1 is not reduced, but slightly increased, indicating that the reduced area of Iba1^+^ cells in the hippocampus of hyperammonemic rats is not due to a decrease in Iba1 expression, but is caused by a change in morphology of microglial cells to a more ameboid shape with shorter prolongations, indicating activated state of microglia (Figure 3E).

**FIGURE 4:**
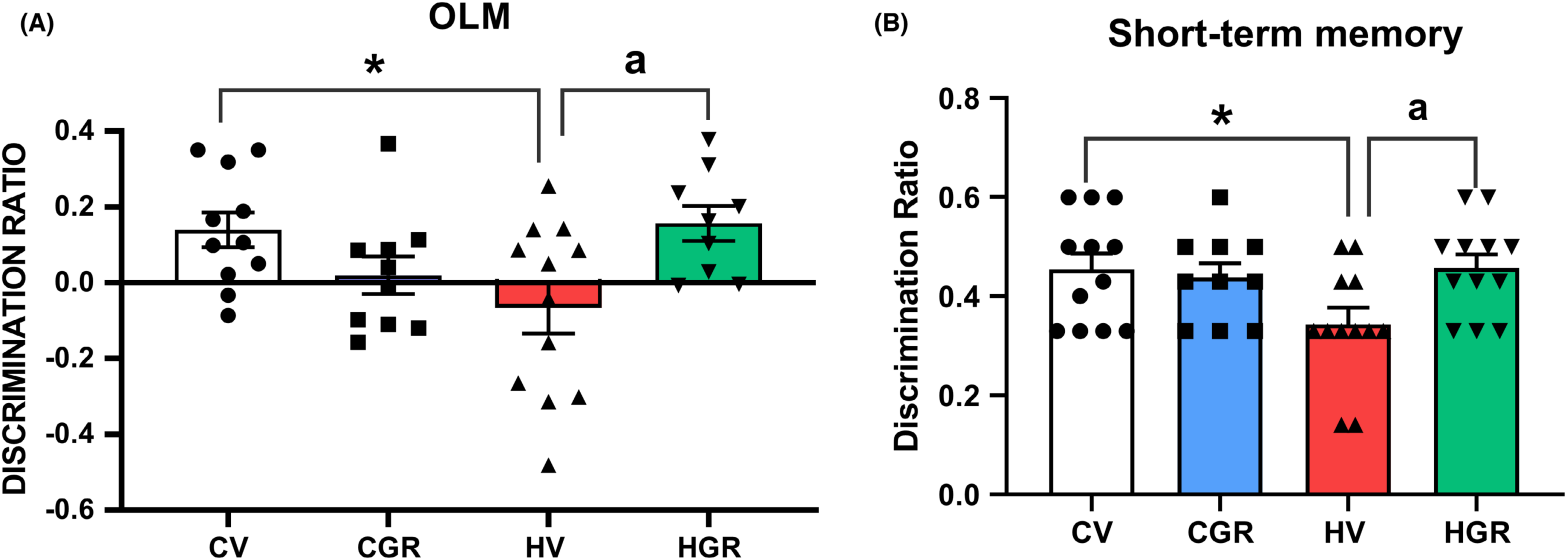
Golexanolone treatment improves novel object location and short‐term memory in hyperammonemic rats. Discrimination ratio was calculated as indicated in methods for novel object location memory (OLM) (A) and for short‐term memory in the Y Maze (B) Values are the mean ± SEM of 10 rats per group for OLM and 11 rats per group for short‐term memory. Values significantly different from control rats are indicated by asterisk and from hyperammonemic rats by “a”. *p < 0.05; ^a^ *p* < 0.05. CGR, control group with golexanolone treatment; CV, control rats with treatment vehicle (CAPMUL); HGR, hyperammonemic rats with golexanolone treatment; HV, hyperammonemic rats with vehicle

We analyzed spatial memory using the novel object location test. The discrimination ratio was reduced in hyperammonemic (−0.067 ± 0.067 vs. 0.14 ± 0.046 in controls) and treatment with golexanolone restored it (0.16 ± 0.046). Short‐term spatial memory in the Y maze was impaired in hyperammonemic rats as reflected in the lower discrimination ratio (0.034 ± 0.034 compared with 0.46 ± 0.028 in controls). Golexanolone reversed the impairment of short‐term memory returning the discrimination ratio to normal values (0.046 ± 0.027) (Figure 4B).

**FIGURE 5:**
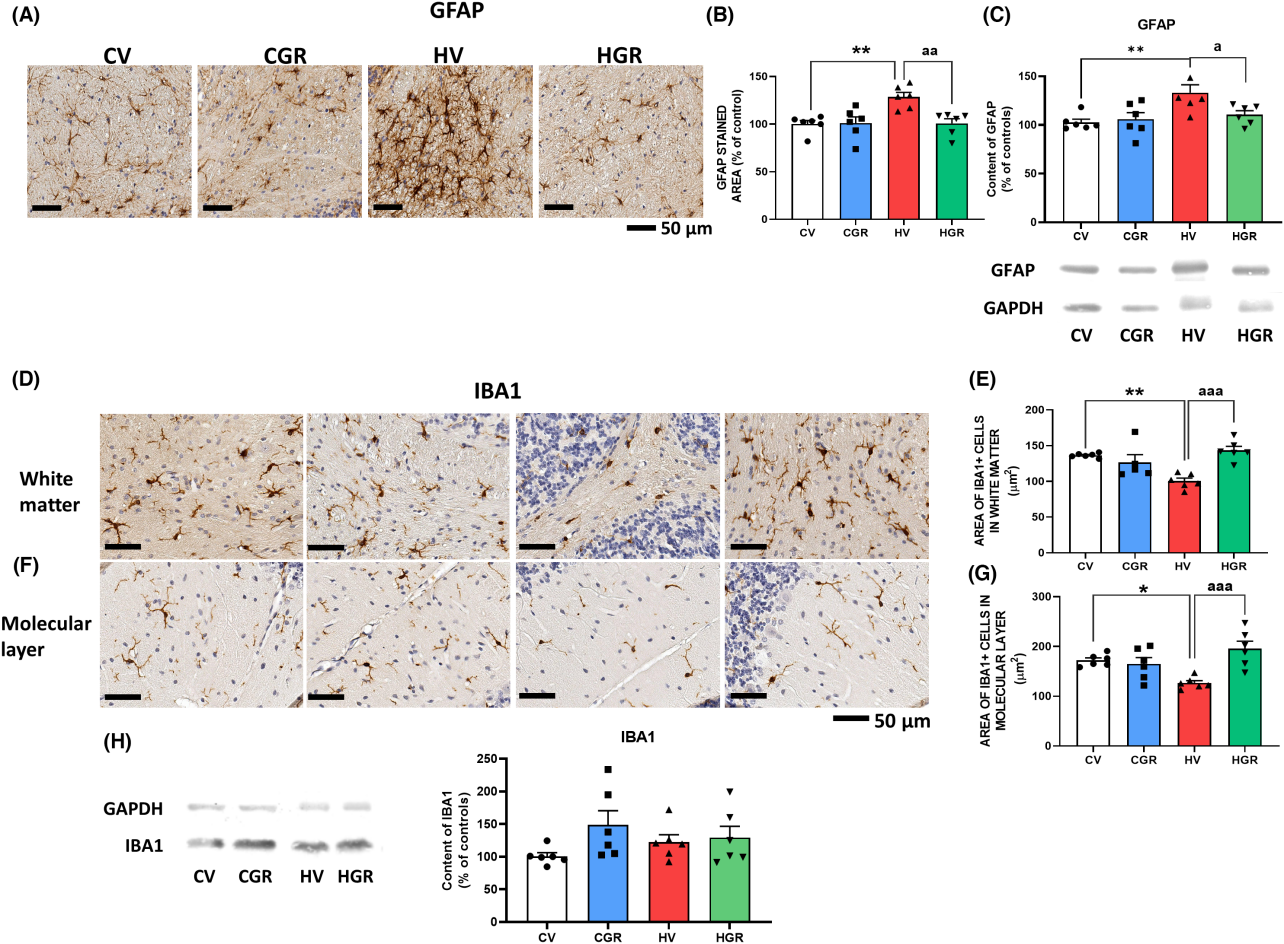
Golexanolone treatment reduces activation of astrocytes and microglia in cerebellum of hyperammonemic rats. Representative immunohistochemistry images of GFAP staining are shown in (A). The area stained by anti‐GFAP was quantified in cerebellum using two slides/rat from 6 rats per group. Values are expressed as percentage of stained area in control rats (B). GFAP content in whole cerebellum was also analyzed by Western blot and expressed as percentage of control rats (C). Representative images of microglia stained with Iba1 in white matter of cerebellum are shown in (D) and in the molecular layer in (F). Microglia activation was quantified by measuring the area of Iba1^+^ cells in white matter (E) and molecular layer (G). Content of Iba1 was analyzed by western blot in whole cerebellar homogenates. Representative bands and quantification is shown in H. Values are the mean ± SEM of 6 rats per group. Values significantly different from control rats are indicated by asterisk and from hyperammonemic rats by “a”. *p < 0.05; **p < 0.01; ^a^ *p* < 0.05; ^aa^ *p* < 0.01 and ^aaa^ *p* < 0.001. CGR, control group with golexanolone treatment; CV, control rats with treatment vehicle (CAPMUL); HGR, hyperammonemic rats with golexanolone treatment; HV, hyperammonemic rats with vehicle

We also analyzed glial activation in cerebellum. Hyperammonemic rats showed astrocytes activation, as shown by immunohistochemistry by the increased (129 ± 5%) area stained by GFAP (Figures 5A,B and S3) and by the increased (130 ± 5%) GFAP content as analyzed by Western blot (Figure 5C). Golexanolone reversed the increase in astrocytes activation, as indicated by the reversal of the increase in GFAP staining (Figure 5A,B) and content (Figure 5C).

Hyperammonemia also increases microglia activation in white matter and in molecular layer of cerebellum, as indicted by the reduced area of microglial cells (101 ± 4 μm^2^ in hyperammonemic rats vs. 137 ± 1 μm^2^ in controls in white matter and 127 ± 5 vs. 172 ± 5 μm^2^ in molecular layer). Golexanolone reversed microglial activation, increasing the area of cells to control values both in white matter (143 ± 6 μm^2^) and molecular layer (196 ± 15 μm^2^) (Figures 5D–G, S4, and S5). As occurs in the hippocampus, the total content of Iba1 in the whole cerebellum as analyzed by Western blot was not reduced (Figure S5H9): The area of Iba1^+^ cells is reduced due to acquisition of a more ameboid shape due to activation (Figure 5H).

**FIGURE 6:**
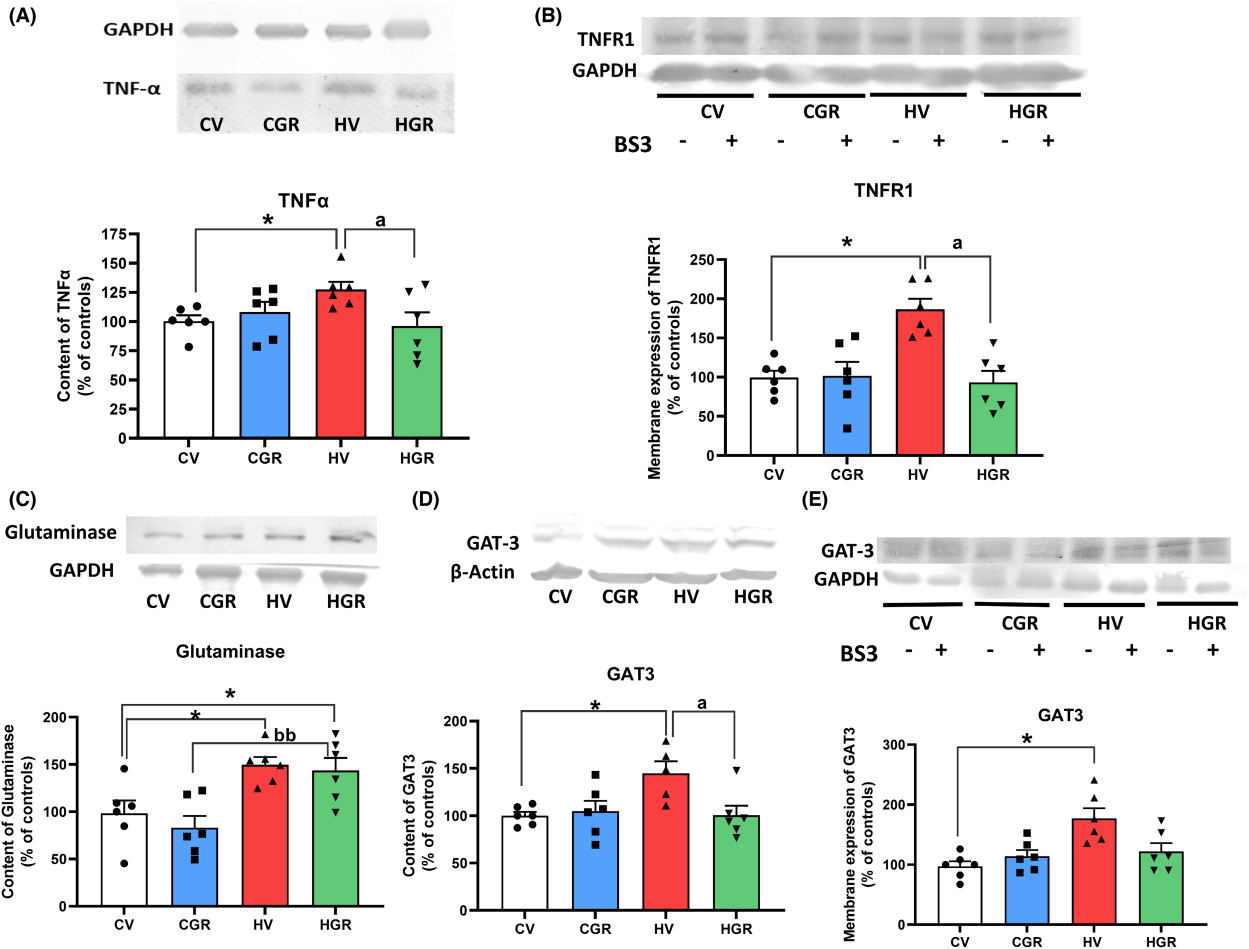
Golexanolone treatment reverses the changes in the TNFα‐TNFR1‐GAT3 pathway induced by hyperammonemia in cerebellum. The content of TNFα (A), glutaminase (C) and GAT3 (D) were analyzed by Western blot. Membrane expression of TNFR1 (B) and of GAT3 (E) were analyzed using BS3 crosslinker and Western blot. Values are the mean ± SEM of 6 rats per group. Values significantly different from control rats are indicated by asterisk and from hyperammonemic rats by “a” and from control rats treated with golexanolone by “b”. *p < 0.05; ^a^ *p* < 0.05; ^bb^ *p* < 0.01. CGR, control group with golexanolone treatment; CV, control rats with treatment vehicle (CAPMUL); HGR, hyperammonemic rats with golexanolone treatment; HV, hyperammonemic rats with vehicle

TNFα levels were increased (134 ± 8%) in cerebellum of hyperammonemic rats and golexanolone reversed this increase, reducing TNFα to normal levels (94 ± 8%) (Figure 6A). Hyperammonemia also increased (189 ± 24%) membrane expression of TNFR1, and golexanolone reversed this increase, returning membrane expression of TNFR1 to control values (88 ± 23%) (Figure 6B).

Glutaminase content was increased (148 ± 11%) in the cerebellum of hyperammonemic rats. Golexanolone did not reduce glutaminase content to normal values, remaining higher than in control rats (139 ± 11%) (Figure 6C).

Hyperammonemia increased the content (148 ± 11%; Figure 6D) and membrane expression (183 ± 20%; Figure 6E) of the GABA transporter GAT3. Golexanolone reversed these increases, returning to normal levels the content (101 ± 10%; Figure 6D) and membrane expression (123 ± 23%; Figure 6E) of GAT3.

**FIGURE 7:**
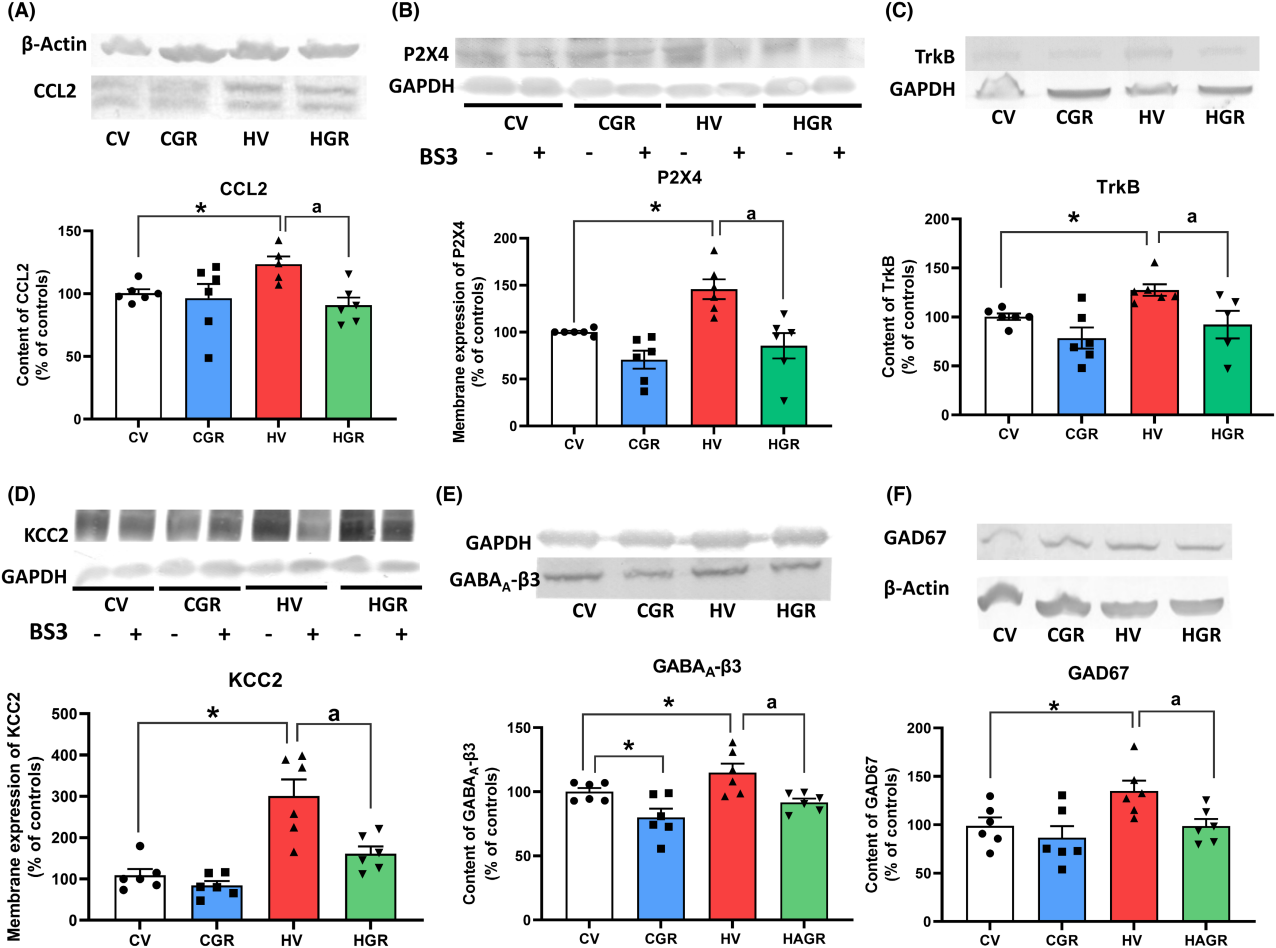
Golexanolone treatment reverses the changes in the TNFR1‐CCL2‐TrkB‐KCC2 pathway and in β3 subunit of GABA_A_ receptors and GAD67 induced by hyperammonemia in cerebellum. The content of CCL2 (A), TrkB (C), β3 subunit of GABA_A_ receptors (E) and GAD67 (F) were analyzed by Western blot. Membrane expression of P2X4 (B) and KCC2 (D) were analyzed using BS3 crosslinker and western blot. Values are the mean ± SEM of 6 rats per group. Values significantly different from control rats are indicated by asterisk and from hyperammonemic rats by “a”. *p < 0.05; ^a^ *p* < 0.05. CGR, control group with golexanolone treatment; CV, control rats with treatment vehicle (CAPMUL); HGR, hyperammonemic rats with golexanolone treatment; HV, hyperammonemic rats with vehicle

We analyzed the TNFR1‐CCL2‐P2X4‐TrkB‐KCC2 pathway. Hyperammonemia increases the levels of CCL2 (121 ± 9%; Figure 7A). Membrane expression of P2X4 was also increased in cerebellum of hyperammonemic rats (148 ± 16% Figure 7B), as well as the content of TrkB (121 ± 5%; Figure 7C) and membrane expression of KCC2 (306 ± 55%; Figure 7D). Treatment with golexanolone reversed these increases (Figure 7A–D).

Hyperammonemia also increased the content of the GABA_A_ receptor subunit β3 (115 ± 6%; Figure 7E) and of the GABA synthesizing enzyme GAD67 (135 ± 14%; Figure 7F). These increases were also reversed by the treatment with golexanolone (Figure 7E–F).

**FIGURE 8:**
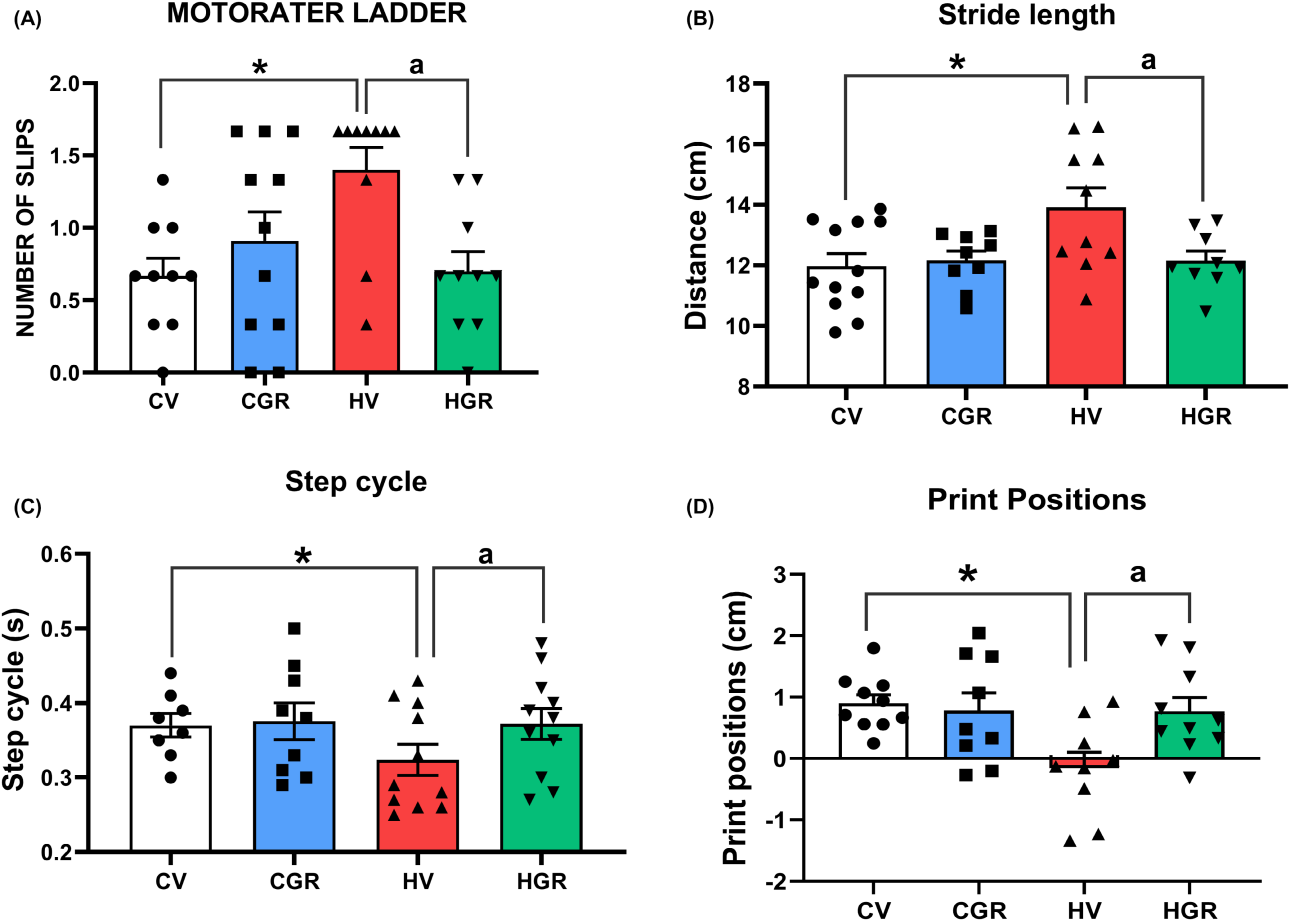
Golexanolone treatment reverses the impairment of motor coordination in the Motorater and motor function in the CatWalk in hyperammonemic rats. Motor coordination was assessed in the Motorater by analyzing wrong foot placements (slips) when the rat crossed a ladder (A). Footprint of locomotor gait was analyzed in the Catwalk™, analyzing the following parameters characterizing locomotor gait: (B) Stride length, (C) Step cycle and (D) Print positions. Values are the mean ± SEM of 11 rats per group in A–C and 9 rats per group in D. Values significantly different from control rats are indicated by asterisk and from hyperammonemic rats by “a”. *p < 0.05; **p < 0.01; ^a^ *p* < 0.05; ^aaaa^ *p* < 0.0001. CGR, control group with golexanolone treatment; CV, control rats with treatment vehicle (CAPMUL); HGR, hyperammonemic rats with golexanolone treatment; HV, hyperammonemic rats with vehicle

These results support the idea that golexanolone reduces GABAergic neurotransmission in cerebellum of hyperammonemic rats by different mechanisms. We assessed if this is associated with improvement of motor coordination and function. Hyperammonemic rats show motor incoordination, with increased number of slips in the motorater (1.4 ± 0.16 slips for hyperammonemic rats compared to 0.67 ± 0.12 slips in controls, *p* = 0.028). Treatment with golexanolone reversed motor incoordination, reducing the number of slips to normal values (0.7 ± 0.14 slips, *p* = 0.038 compared with hyperammonemic rats without treatment) (Figure 8A).

Motor function was also assessed in the CatWalk test, which allows an accurate analysis of locomotor gait and detection of different types of gait alterations that reflect impaired fine motor control, modulated by cerebellum. Hyperammonemic rats show increased stride length (13.5 ± 0.07 cm in hyperammonemic rats, compared to 12 ± 0.09 cm in controls, *p* < 0.0001) which is reversed by golexanolone (12.4 ± 0.06 cm, *p* = 0.0097 compared with non‐treated hyperammonemic rats) (Figure 8B). Other parameters such as step cycle (Figure 8C) and print positions (Figure 8D) show reduced values in hyperammonemic rats and were normalized by golexanolone (Figure 8C,D). Other parameters of gait affected in hyperammonemia and the effects on them of golexanolone are shown in Table 1.

**TABLE 1:** Analysis of gait and fine motor coordination parameters in the CatWalk

	CV	CGR	HV	HGR
*Paw statistic parameters*				
Print area hind paws				
Right	1.90 ± 0.11	1.93 ± 0.05	1.70 ± 0.08	2.01 ± 0.08
Left	1.98 ± 0.11	1.89 ± 0.11	1.72 ± 0.05	1.96 ± 0.07
Stand index hind paws				
Right	−5.0 ± 0.4	−4.2 ± 0.1	−6.9 ± 1.0	−4.5 ± 0.5^a^
Left	−4.1 ± 0.4	−3.9 ± 0.2	−6.6 ± 0.8*	−4.1 ± 0.6^a^
Swing %				
Right front	35.7 ± 0.9	35.0 ± 1.3	40.9 ± 1.1*	38.0 ± 0.9
Right hind	31.1 ± 1.2	31.0 ± 1.3	36.5 ± 1.7*	32.8 ± 1.0
Left front	36.6 ± 0.9	34.1 ± 1.6	41.2 ± 1.1*	38.8 ± 1.0
Left hind	29.9 ± 1.5	30.1 ± 1.2	35.9 ± 1.7**	32.1 ± 1.2
Dual stance hind				
Initial right	0.074 ± 0.009	0.065 ± 0.010	0.048 ± 0.010	0.079 ± 0.011
Initial left	0.078 ± 0.013	0.078 ± 0.011	0.045 ± 0.008	0.064 ± 0.008
Terminal right	0.076 ± 0.012	0.074 ± 0.010	0.043 ± 0.007	0.077 ± 0.010
Terminal left	0.077 ± 0.011	0.067 ± 0.012	0.041 ± 0.008	0.074 ± 0.011
*Step sequence parameters*				
Base of support hind paws				
	3.8 ± 0.1	3.7 ± 0.09	3.4 ± 0.05*	3.3 ± 0.08**
Regularity index				
	99.1 ± 0.4	98.1 ± 0.7	97.6 ± 0.8	99.2 ± 0.4

*Note*: Footprint of gait in rats was analyzed in the Catwalk™, analyzing different parameters characterizing locomotor gait, both related to paw statistics and of step sequence, the last assessing fine motor coordination. Values are the mean ± SEM of 9 rats per group. Statistical analysis with One‐way ANOVA brought the following data: *F*(3, 72) = 3.799; *p* = 0.0138 for *Print area of hind* paws; *F*(3, 164) = 6.741; *p* = 0.0003 for *Stand Index of hind* paws; *F*(3, 156) = 18.7; *p* < 0.0001 for *Swing*; *F*(3, 76) = 3.479; *p* = 0.0199 for *Initial dual stance of hind paws* and *F*(3, 77) = 5.235; *p* = 0.0024 for *Terminal dual stance of hind* paws. For *Base of support of hind paws* statistic values were *F*(3, 40) = 6.209; *p* = 0.0015 and for *Regularity index F*(3, 22) = 1.902; *p* = 0.1587, analyzed with Brown‐Forsythe ANOVA test. Post‐hoc analysis with Tukey's Multiple comparisons test brought the statistic differences indicated in the table. Values significantly different from control rats are indicated by asterisk and from hyperammonemic rats by “a”.

Abbreviations: CV, control rats with treatment vehicle (CAPMUL); CGR, control group with golexanolone treatment; HV, hyperammonemic rats with vehicle; HGR, hyperammonemic rats with golexanolone treatment.

* *p* < 0.05

** *p* < 0.01;

^a^ *p* < 0.05.

## 4 DISCUSSION

The results reported show that treatment with golexanolone reduces peripheral inflammation and reverses the activation of microglia and astrocytes and neuroinflammation in hippocampus and cerebellum of hyperammonemic rats. This is associated with reversal of cognitive impairment and of the alterations in motor function and coordination. Moreover, we unveil some mechanisms by which this reduction in neuroinflammation by golexanolone may contribute to reduce GABAergic neurotransmission, leading to improved cognitive and motor function.

Golexanolone is a GABA_A_ receptor modulating steroid antagonist that reduces GABAergic neurotransmission by reducing the potentiation by allopregnanolone of GABA_A_ receptors activation.^27^, ^38^, ^39^, ^40^ However, we show here that this is not the only mechanism by which golexanolone may reduce the enhanced GABAergic neurotransmission in hyperammonemic rats and improve neurological function.

Golexanolone reduces peripheral inflammation in hyperammonemic rats, normalizing TNFα and IL‐10 levels. Chronic hyperammonemia is enough to induce peripheral inflammation, which mediates the induction of neuroinflammation in hippocampus and cognitive impairment.^15^ Treatment of hyperammonemic rats with bicuculline, an antagonist of GABA_A_ receptors, also normalizes plasma levels of TNFα and IL‐10.^21^ We show here that reducing GABAergic neurotransmission with golexanolone also reverses peripheral inflammation in hyperammonemic rats. The underlying mechanisms remain unclear. Intracerebral administration of bicuculline reverses the increase in serum TNFα levels induced by i.v. lipopolysaccharide (LPS) injection in rats,^41^ suggesting a role for cerebral GABA_A_ receptors in the reduction of inflammation by bicuculline. However, GABA_A_ receptors in cells of the immune system may also mediate the immunomodulatory effects of GABA. Both proinflammatory and antiinflammatory effects of GABA and of GABA_A_ receptors have been proposed,^42^, ^43^, ^44^, ^45^, ^46^, ^47^, ^48^, ^49^ suggesting that GABAergic components are a new therapeutic approach for inflammatory and autoimmune diseases.^50^ The results reported here show that treatment with golexanolone reverses peripheral inflammation in hyperammonemic rats and could be beneficial in certain inflammatory or autoimmune diseases.

We show here that golexanolone reduces both peripheral inflammation and neuroinflammation, thus inducing antiinflammatory effects. Antiinflammatory effects have been also reported for allopregnanolone, which reduces the proinflammatory signaling induced by activation of TLR4.^51^ However, these effects are independent of GABA_A_ receptors, and are also induced by pregnenolone, which does not potentiate GABA_A_ receptors activation, and are due to the steroid D ring. This antiinflammatory effect would be due to interference by allopregnanolone of MyD88 binding to TLRs.^52^ Allopregnanolone reverses neurogenic and cognitive deficits in mouse models of Alzheimer's disease.^53^, ^54^ These effects are mediated by activation of GABA_A_ receptors^53^, ^55^ indicating that they are not due to the antiinflammatory effect of allopregnanolone.^51^, ^52^

Allopregnanolone induces biphasic effects,^56^ depending on the persistence of the increase of allopregnanolone, single or intermittent exposure‐induced beneficial effects on neurogenesis and learning,^56^ while chronically elevated allopregnanolone levels impaired learning.^57^, ^58^, ^59^ These effects would be a consequence of the persistence of the activation and do not seem to depend on the concentration of allopregnanolone, as supported by the data on Table 1 in Ref. [55] summarizing many studies using chronic or acute administration of different doses of allopregnanolone^56^ Allopregnanolone levels are increased in brain of cirrhotic patients who died in hepatic coma,^26^ and in rats with hepatic encephalopathy due to portacaval shunts.^60^ Treatment of these rats with indomethacin reduces allopregnanolone in brain and improves locomotor deficits.^60^ Allopregnanolone is also increased in brain of hyperammonemic rats.^25^ Sustained overactivation of GABA_A_ receptors by allopreganolone in hyperammonemia and hepatic encephalopathy would contribute to the enhanced GABAergic tone and to the associated cognitive and motor deficits. Reducing this overactivation with golexanolone would be a useful therapeutic approach to reverse cognitive and motor impairment in these pathological situations. We show that golexanolone reverses activation of microglia and astrocytes and neuroinflammation in hippocampus and cerebellum of hyperammonemic rats. The reversal of peripheral inflammation may contribute to this reversal of neuroinflammation, but direct effects of golexanolone would also contribute.

There is an interplay between GABAergic neurotransmission and neuroinflammation which modulate each other and contribute to the induction of cognitive and motor impairment.^14^, ^29^ The reversal of neuroinflammation by golexanolone would be a consequence of the reduction of GABAergic neurotransmission, as occurs for bicuculline.^21^, ^23^ The primary effect of golexanolone would be a reduction of potentiation of GABA_A_ receptors activation by allopregnanolone,^27^ thus reducing GABAergic neurotransmission and, subsequently, neuroinflammation.

Neuroinflammation, in turn, enhances GABAergic neurotransmission. Neuroinflammation increases TNFα and its receptor TNFR1. Enhanced activation of TNFR1 increases GABAergic neurotransmission in cerebellum of hyperammonemic rats by activating the TNFR1‐NF‐kB‐glutaminase‐GAT3 pathway^14^, ^20^ and the TNFR1‐CCL2‐TrkB‐KCC2 pathway.^19^ Enhanced activation of CCR2 by CCL2 contributes to altered membrane expression of KCC2 and NKCC1, leading to enhanced GABAergic neurotransmission in cerebellum of hyperammonemic rats.^19^ Enhanced activation of TNFR1 induces activation of the transcription factor NF‐kB, which increases the levels of glutaminase, leading to increased extracellular glutamate. Enhanced uptake of glutamate and Na^+^ by glutamate transporters in activated astrocytes alters the transmembrane Na^+^ gradient leading to reversal of the GABA transporter GAT3 function, which releases GABA to the extracellular fluid. Moreover, hyperammonemia increases membrane expression of GAT3, further increasing extracellular GABA and contributing to motor incoordination.^14^, ^20^, ^61^

We show here that golexanolone reverses the activation of this pathway in cerebellum of hyperammonemic rats, reducing to normal values the TNFα content, membrane expression of TNFR1 and the content and membrane expression of GAT3. This would reverse the increase in extracellular GABA and its contribution to enhanced GABAergic neurotransmission.

Another mechanism by which increased activation of TNFR1 enhances GABAergic neurotransmission is by activating the TNFR1‐CCL2‐TrkB‐KCC2 pathway. Enhanced activation of TNFR1 increases CCL2 content, which contributes to activate microglia and increase BDNF, which activates TrkB that increases membrane expression of the chloride co‐transporter KCC2. KCC2 is expressed in neurons and extrudes chloride ion from the neuron increasing the chloride gradient, hyperpolarizing the neuron and increasing the responses to activation of GABA_A_ receptors by allopregnanolone and GABA.^19^, ^62^, ^63^, ^64^

We show here that, in cerebellum of hyperammonemic rats, golexanolone also reverses the overactivation of the TNFR1‐CCL2‐TrkB‐KCC2 pathway, which would normalize the transmembrane chloride gradient, contributing to reduce GABAergic neurotransmission.

Golexanolone also reduces GABAergic neurotransmission by reversing the increase of the GABA synthesizing GAD67, which would reduce GABA concentration, and by reversing the increased membrane expression of the β3 subunit of GABA_A_ receptors, which would reduce its activation.

The reversal of neuroinflammation and of enhanced GABAergic neurotransmission by golexanolone treatment of hyperammonemic rats is associated with concomitant improvement of the impaired cognitive function, motor function and coordination. A similar improvement of cognitive and motor function would be expected to be achieved by golexanolone treatment in cirrhotic patients with minimal or clinical HE. These patients also show neuroinflammation in cerebellum, with microglia and astrocytes activation^65^ enhanced GABAergic neurotransmission in cerebellum,^66^ and impaired cognitive function and motor function and coordination.^1^, ^2^, ^3^, ^4^, ^7^, ^12^, ^67^ In addition to the expected effects in HE, golexanolone may also have beneficial effects in improving neurological function in patients with other pathologies associated with neuroinflammation and enhanced GABAergic neurotransmission. It has been shown that oral administration of golexanolone (GR3027) mitigates inhibition of brain function induced by allopregnanolone in healthy adult males at doses which are clinically well tolerated.^39^ Moreover, safety, pharmacokinetics and efficacy of golexanolone has been investigated in adult patients with cirrhosis with promising results. Golexanolone exhibited satisfactory safety and pharmacokinetic, was well tolerated and associated with improvement in cognitive performance.^28^ These studies, together with the data reported here support the therapeutic potential of golexanolone.

## 5 CONCLUSION

Reducing GABA_A_ receptors activation with golexanolone reduces peripheral inflammation and neuroinflammation and improves cognitive and motor function in hyperammonemic rats. The mechanistic and therapeutic effects identified would also occur in patients with hepatic encephalopathy and, likely, in other pathologies associated with neuroinflammation.

## CONFLICT OF INTEREST

This study was financed by Umecrine Cognition AB, which is developing GR3027/Golexanolone.

## Supporting information


**FIGURE S1** Representative images with different magnifications of GFAP staining in hippocampus, corresponding to astrocytes activation results presented at main Figure 3A.

**FIGURE S2** Representative images with different magnifications of Iba1 staining in hippocampus, corresponding to microglia activation results presented at main Figure 3C.

**FIGURE S3** Representative images with different magnifications of GFAP staining in white matter of cerebellum, corresponding to astrocytes activation results presented at main Figure 5A.

**FIGURE S4** Representative images with different magnifications of Iba1 staining in white matter of cerebellum, corresponding to microglia activation results presented at main Figure 5D.

**FIGURE S5** Representative images with different magnifications of Iba1 staining in molecular layer of cerebellum, corresponding to microglia activation results presented at main Figure 5F.

## Data Availability

The data that support the findings of this study are available from the corresponding author upon reasonable request.
