# Supplementary figures and images for: Golexanolone, a GABAA receptor modulating steroid antagonist, restores motor coordination and cognitive function in hyperammonemic rats by dual effects on peripheral inflammation and neuroinflammation
Source: CNS Neurosci Ther. 2022 Jul 26;28(11):1861–74. doi: 10.1111/cns.13926 (PMC9532914; doi:10.1111/cns.13926)

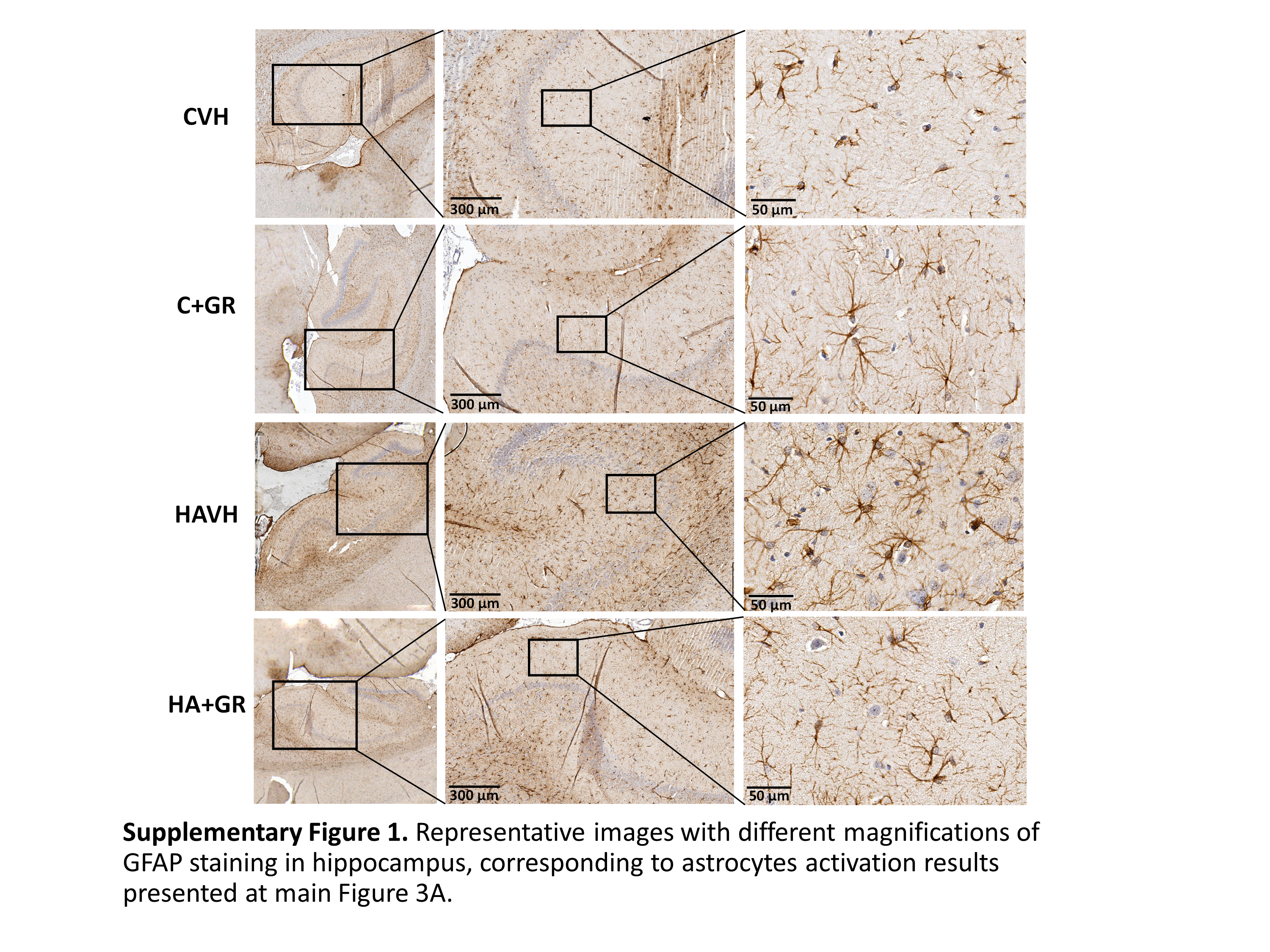

Supplement: Supplementary file 1 — FIGURE S1 Representative images with different magnifications of GFAP staining in hippocampus, corresponding to astrocytes activation results presented at main Figure 3A. [file CNS-28-1861-s003.TIF]

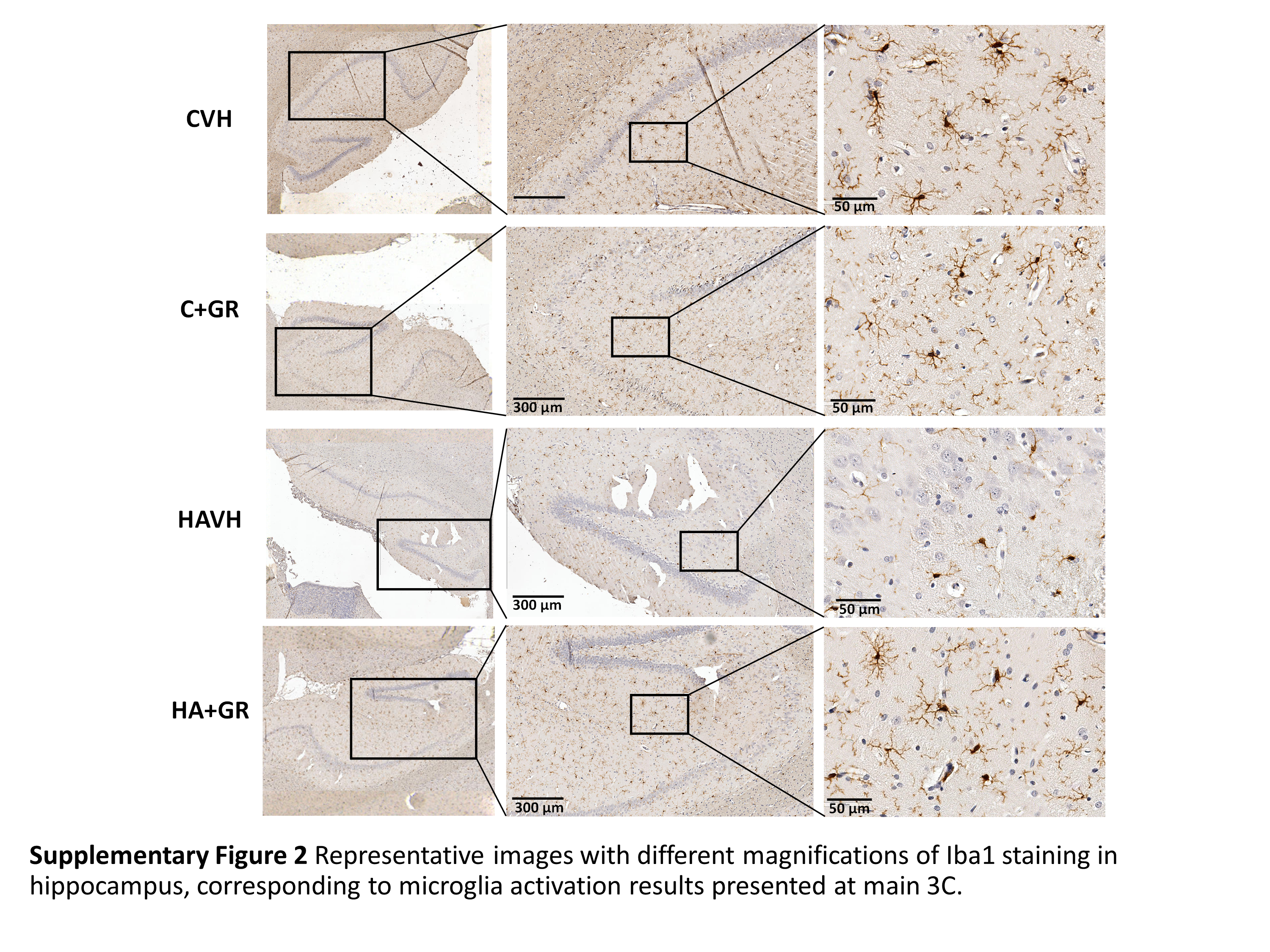

Supplement: Supplementary file 2 — FIGURE S2 Representative images with different magnifications of Iba1 staining in hippocampus, corresponding to microglia activation results presented at main Figure 3C. [file CNS-28-1861-s002.TIF]

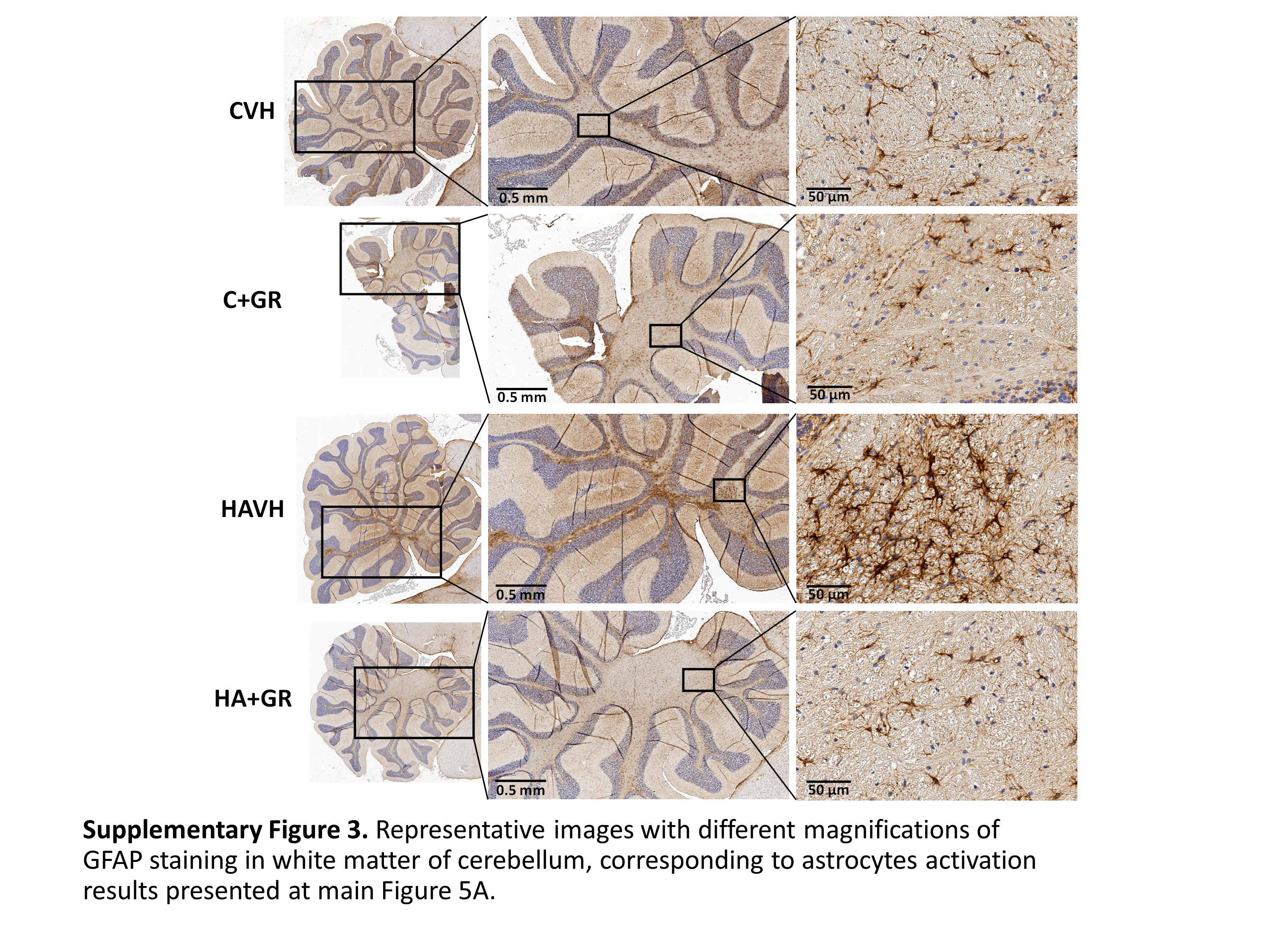

Supplement: Supplementary file 3 — FIGURE S3 Representative images with different magnifications of GFAP staining in white matter of cerebellum, corresponding to astrocytes activation results presented at main Figure 5A. [file CNS-28-1861-s004.TIF]

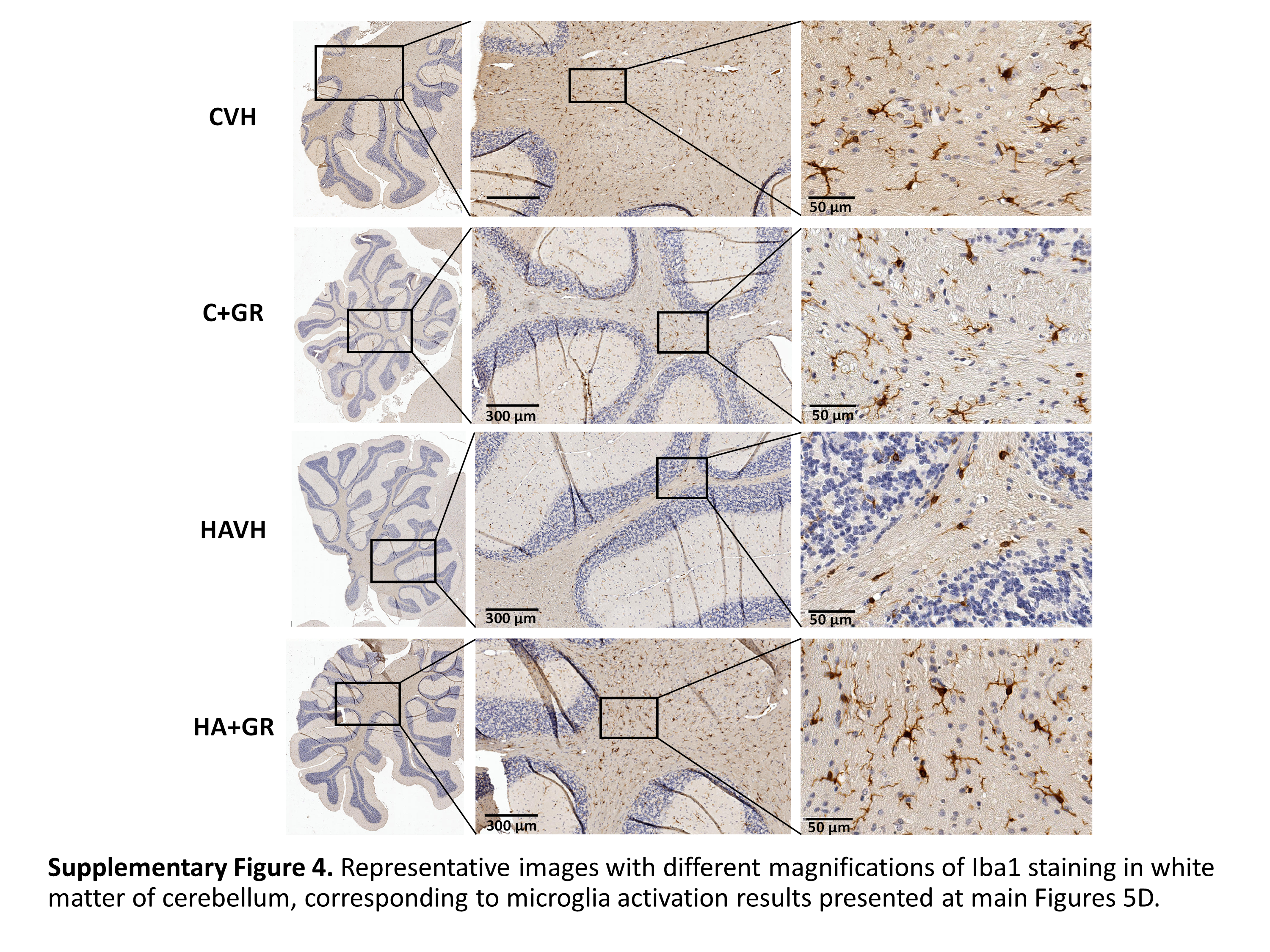

Supplement: Supplementary file 4 — FIGURE S4 Representative images with different magnifications of Iba1 staining in white matter of cerebellum, corresponding to microglia activation results presented at main Figure 5D. [file CNS-28-1861-s001.TIF]

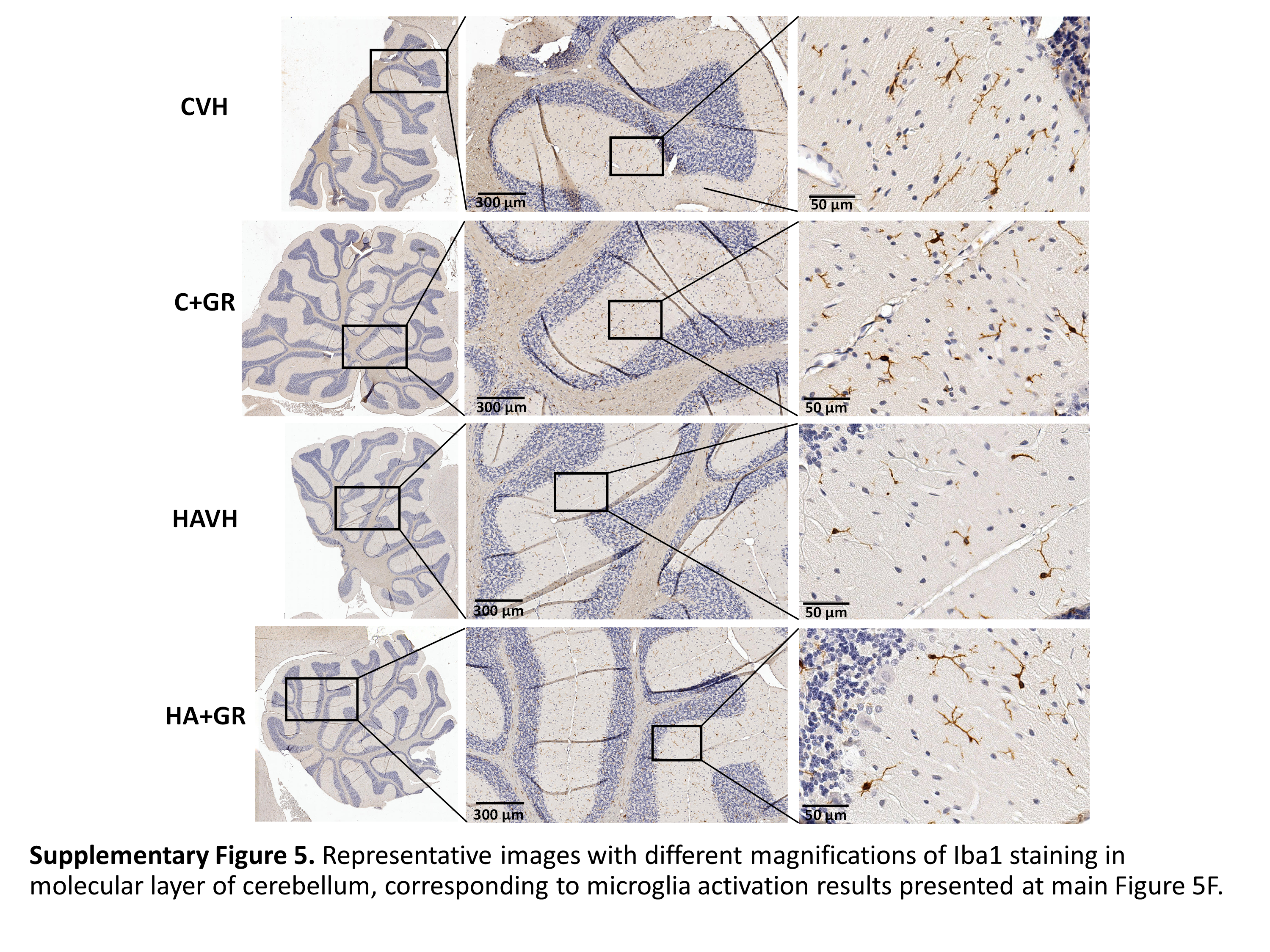

Supplement: Supplementary file 5 — FIGURE S5 Representative images with different magnifications of Iba1 staining in molecular layer of cerebellum, corresponding to microglia activation results presented at main Figure 5F. [file CNS-28-1861-s005.TIF]
